# Asymptotic behavior of HIV-1 epidemic model with infinite distributed intracellular delays

**DOI:** 10.1186/s40064-016-1951-9

**Published:** 2016-03-12

**Authors:** Nigar Ali, Gul Zaman

**Affiliations:** Department of Mathematics, University of Malakand, Chakdara Dir (Lower), Khyber Pakhtunkhawa Pakistan

**Keywords:** HIV-1 epidemic model, Distributed delay, Unique positive solution, Stability analysis, 92D25, 49J15, 93D20, 91G20

## Abstract

In this study, asymptotic analysis of an HIV-1 epidemic model with distributed intracellular delays is proposed. One delay term represents the latent period which is the time when the target cells are contacted by the virus particles and the time the contacted cells become actively infected and the second delay term represents the virus production period which is the time when the new virions are created within the cell and are released from the cell. The infection free equilibrium and the chronic-infection equilibrium have been shown to be locally asymptotically stable by using Rouths Hurwiths criterion and general theory of delay differential equations. Similarly, by using Lyapunov functionals and LaSalle’s invariance principle, it is proved that if the basic reproduction ratio is less than unity, then the infection-free equilibrium is globally asymptotically stable, and if the basic reproduction ratio is greater than unity, the chronic-infection equilibrium is globally asymptotically stable. Finally, numerical results with conclusion are discussed.

## Background

Human immunodeficiency virus (HIV-1) is a lentivirus that causes acquired immunodeficiency syndrome (AIDS). The HIV-1 infection passes through three different phases, viz, the primary infection, chronic infection and acquired immunodeficiency syndrome (AIDS). In the primary infection, viral load experiences, a substantial increase to the peak level, followed by decline to the steady state, which is referred to as the viral set point. To control this infection, many scientists and researchers have been focusing on it, as there is no effective way to cure AIDS.

In the recent research, recombinant virus is used for controlling the infection of HIV-1 (see for example, Wagner and Hewlett 1999; Nolan 1997). Revilla and Garcya-Ramos (2003) established a 5-dimensional ordinary differential equation system to investigate the control of the infection by introducing a recombinant virus to fight the virus. Jiang et al. (2009), introduced a constant injection rate of the recombinant virus and presented various bifurcation patterns. A control strategy of the HIV-1 epidemic model was given in Yu and Zou (2012). The following differential equations are standard and classic in-host model for HIV-1 infection:

1 $$\begin{aligned} \dot{x}(t) &= \lambda -d x(t)-\beta x(t) v(t), \\ \dot{y}(t) &=  \beta x(t) v(t)-ay(t), \\ \dot{v}(t) &= k y(t)-p v(t),  \end{aligned}$$

where *x*(*t*), *y*(*t*) and *v*(*t*) represent the densities of uninfected cells, infected cells and the free virus cells, respectively at time *t*. $\lambda$ represents the rate at which new target cells are generated, *d* is the specific death rate and $\beta$ is the constant rate at which a T-cell is contacted by the virus. It is assumed that once cells are infected, they may die at rate *a* either due to the virus or the immune system, and in the mean time, each cell produces new virus particles at a rate *k* during their life, *p* is the death rate of virus cells.

Revilla and Garcya-Ramos (2003) extended the model (1) by adding a second virus which may cause the infected cells to have a second infection, called double-infection, leading to a modified model is given by

2 $$\begin{aligned} \dot{x}(t) &= \lambda -d x(t)-\beta x(t) v(t), \\ \dot{y}(t) &= \beta x(t) v(t)-a y(t)-\alpha w(t)y(t), \\ \dot{z}(t) &= a w(t)y(t)-b z(t),\\ \dot{v}(t) &= k y(t)-p v(t), \\ \dot{w}(t) &= c z(t)-q w(t).  \end{aligned}$$

Here *w*(*t*) and *z*(*t*) represent genetically modified(recombinant) virus and double-infected cells, respectively. It is assumed that recombinant infected cells previously infected by the pathogen virus and turn them at rate $\alpha$ into doubly-infected cells. In the mean time recombinant are removed at a rate *q*. The doubly infected cells die at a rate of *b* and release recombinant at a rate *c*. In Revilla and Garcya-Ramos (2003), the authors analyzed the structure of equilibrium solutions and presented some simulations of the model (2). The global attractivity of the three concerned disease-free equilibria is presented in Shang (2015) by using Lyapunov functional theory. Jiang et al. (2009), presented the stability of all possible equilibrium solutions and bifurcations between these equilibria, as well as proved the existence of Hopf bifurcation. Yu and Zou (2012), modified the model (2) by incorporating a control parameter $\eta$ to measure the injection rate of the recombinant for controlling/eliminating the HIV virus. Tian et al. (2014) introduced the time lag into the model (2). Since in real situation, time is needed for the virus to contact a target cell and then the contacted cells become actively affected. Keeping in view this time lag they modified the model (2), by using the idea of Zhu and Zou (2008, 2009), as follows

3 $$\begin{aligned} \dot{x}(t) &=  \lambda -d x(t)-\beta x(t) v(t), \\ \dot{y}(t) & = \beta e^{-a\tau } x(t-\tau ) v(t-\tau )-a y(t)-\alpha w(t)v(t), \\ \dot{z}(t)& = a w(t)y(t)-b z(t),\\ \dot{v}(t)& = k y(t)-p v(t), \\ \dot{w}(t)& = c z(t)-q w(t), \end{aligned}$$

where $\tau$ denotes the average time for a viral particle to go through the eclipse phase. Here *a* is the constant death rate for infected cells but which are not virus producing cells yet. Therefore, $e^{-a\tau }$ is the probability of surviving in the time period from $t-\tau$ to *t*. The effect of intracellular delays on viral infection has been discussed in Culshaw et al. (2003), Herz et al. (1996), Mittler et al. (1999), Nelson et al. (2000), Nelson and Perelson (2002), Xu (2011), while the effect of different awareness campaign on the spread of random network has been investigated in Shang (2013).

This paper focuses on the dynamical behavior of the system with delays and studies their equilibrium solutions with bifurcations. This study extends the work presented in Tian et al. (2014) by incorporating two distributed intracellular delays. In the proposed model one delay term represents the latent period which is the time that the target cells are contacted by the virus particles and the time the contacted cells become actively infected. While the second delay term represents the virus production period which means the time during when new virions are created within the cell and are released from the cell. The proposed model becomes

4 $$\begin{aligned} \dot{x}(t)& = \lambda -d x(t)-(1-n_{rt})\beta x(t) v(t), \\ \dot{y}(t)& = (1-n_{rt})\beta \int _{0}^{\infty } e^{-m\tau }f_{1}(\tau ) x(t-\tau ) v(t-\tau )d\tau -a y(t)-\alpha w(t)y(t), \\ \dot{z}(t)& = a w(t)y(t)-b z(t),\\ \dot{v}(t)& = (1-n_{p})k \int _{0}^{\infty }f_{2}(\tau )y(t-\tau )d\tau -p v(t), \\ \dot{w}(t)& = c z(t)-q w(t). \end{aligned}$$

The measure of the efficacies of the protease inhibitor and the reverse transcriptase inhibitor are denoted by $$n_{p}$$ and $$n_{rt}$$, respectively. It is also assumed that the infected cells become productively infected $$\tau $$ units later, where $$\tau $$ is distributed according to the probability distribution $$f_{1}(\tau )$$. The recruitment of virus producing cells at time *t* is given by the number of the cells that were infected at time $$t-\tau $$ and are still alive at time *t*. Here *m* is the constant death rate for infected cells but which are not virus producing cells as yet. Therefore, $$e^{-m\tau }$$ is the probability of surviving in the time period from $$t-\tau $$ to *t*. Also it is assumed that $$\tau $$ units later the virus penetrated into the cell at time *t*, where $$\tau $$ is distributed according to the probability distribution $$f_{2}(\tau )$$. The probability distribution functions like $$f_{1}(\tau )=\delta (t-\tau _{1}), $$$$f_{2}(\tau )=\delta (\tau )$$ or $$f_{1}(\tau )=\delta (\tau )$$, $$f_{2}(\tau )=\delta (t-\tau _{2})$$, are proposed. After some manipulation our proposed model in general form can be written as follows:

5 $$\begin{aligned} \dot{x}(t)& = \lambda -d x(t)-\widetilde{\beta } x(t) v(t), \\ \dot{y}(t)& = \widetilde{\beta }\int _{0}^{\infty } e^{-m_{1}\tau }f_{1}(\tau ) x(t-\tau ) v(t-\tau )d\tau -a y(t)-\alpha w(t)y(t), \\ \dot{z}(t)& = a w(t)y(t)-b z(t),\\ \dot{v}(t)& = \widetilde{k} \int _{0}^{\infty } e^{-m_{2}\tau }f_{2}(\tau )y(t-\tau )d\tau -p v(t), \\ \dot{w}(t)& = c z(t)-q w(t), \end{aligned}$$

where $\widetilde{\beta }=(1-n_{rt})\beta$ and $\widetilde{k}=(1-n_{p})k$. The term $e^{-m_{2}\tau }$ is the probability of surviving from time $t-\tau$ to time *t*, where $m_{2}$ is the death rate of infected but not yet virus-producing cells. In the system (5), the delay kernel is assumed to be piecewise continuous to satisfy $\int _{0}^{\infty }f_{i}(\tau )d\tau = 1$ and $\int _{0}^{\infty }\tau f_{i}(\tau )d\tau < 1, i=1,2$. The initial conditions for the system (5) become

6 $$ x(\zeta )=\psi _{1}(\zeta ), y(\zeta )=\psi _{2}(\zeta ), z(\zeta )=\psi _{3}(\zeta ),v(\zeta )=\psi _{4}(\zeta ), w(\zeta )=\psi _{5}(\zeta ), \zeta \in [-\infty , 0].$$

Here $(\psi _{1},\psi _{2},\psi _{3},\psi _{4},\psi _{5})\in C[(-\infty ,0), R^{5}]$ be the space of continuous functions mapping the interval $(-\infty ,0]$ into $R^{5}$, where $\psi _{i}(\zeta )\ge 0, i=1,2,,\ldots ,5$ and $R^{5}=\{(x_{1},x_{2},x_{3},x_{4},x_{5}); x_{i}\ge 0,i=1,2,\ldots ,5\}$.

According to the fundamental theory of functional differential equations (Kuang 1993), the system (5) admits a unique solution of (*x*(*t*), *y*(*t*), *v*(*t*), *z*(*t*), *w*(*t*)) and satisfies the initial conditions (6). It is easy to show that all solutions of the system (5) with initial conditions (6) are defined on $[0, +\infty )$ and remain positive for all $t \ge 0$.

The rest of the paper is organized as follows: In “Positivity and well-posdeness of the solution” section, we address the well-posedness of the model by proving the positivity and boundedness of solutions. We also identify the basic reproduction number $R_{0}$ which determines whether there is or not an uninfected equilibrium. In “Local behavior of the proposed model” section, local stability has been discussed and it is proved that disease free equilibrium is locally stable if $R_{0}<1$ and chronic-infection equilibrium is locally stable if $R_{0}>1$. “Global behavior of the proposed model” section is dedicated to the global stability of the proposed model. Numerical simulations and discussion are presented in “Numerical simulation” section. Finally, conclusion is given in “Conclusion and discussion” section.

## Positivity and well-posdeness of the solution

In this section, we will discuss positivity and boundedness of the solution. The following theorem gives boundedness and positivity of the solution.

### **Theorem 1**

*All solutions of the system* (5) *remain non-negative, provided the given conditions are non-negative and bounded.*

### *Proof 1*

By using variation of parameter formulae, we get the following solution of the system (5)

$$\begin{aligned} x(t)& = x(0)e^{- \int _0^t (d+\widetilde{\beta } v(\zeta ))d\zeta } +\lambda \int _0^t e^{- \int _{\eta }^t (d+\widetilde{\beta } v(\zeta ))d\zeta } d\eta , \\ y(t)& = y(0)e^{- \int _0^t (a+\alpha w(\zeta ))d\zeta } +\widetilde{\beta } \int _0^t e^{- \int _{\eta }^t (a+\alpha w(\zeta ))d\zeta }\int _{0}^{\infty } e^{-m_{1}\tau }f_{1}(\tau ) x(t-\tau ) v(t-\tau ) d\tau d\eta ,\\ z(t)& = z(0)e^{-b t} + \int _0^t \alpha w(t) y(t)e^{- \int _{\eta }^t b(t-\zeta ) d\zeta }d\eta ,\\ v(t)& = v(0)e^{-p t} +\widetilde{k} \int _0^t e^{-p(t-\eta )} \int _{0}^{\infty } e^{-m_{2}\tau }f_{2}(\tau )y(t-\tau )d\tau d\eta ,\\ w(t)& = w(0)e^{-\widetilde{k} t} + c\int _0^t z(\eta )e^{-\widetilde{k}(t-\eta )}d\eta . \end{aligned}$$

Which shows the positivity of the solution of each solution *x*(*t*), *y*(*t*), *v*(*t*), *z*(*t*) and *w*(*t*).

Next, we show the boundedness of the solution. We define

$$\begin{aligned} D(t)& = c\widetilde{k}\int _{0}^{\infty } e^{-m_{2}\tau }f_{2}(\tau )\int _{0}^{\infty } e^{-m_{1}\tau }f_{1}(\tau ) x(t-\tau )d\tau d\tau + c\widetilde{k} \int _{0}^{\infty } e^{-m_{2}\tau }f_{2}(\tau )y(t)d\tau \\&\quad+\,c\tilde{k}\int _{0}^{\infty } e^{-m_{2}\tau }f_{2}(\tau ) z(t)d\tau + \frac{ac}{2} v(t+\tau ) +\frac{b\widetilde{k}}{2}\int _{0}^{\infty } e^{-m_{2}\tau }f_{2}(\tau ) w(t)d\tau . \end{aligned}$$

Calculating the derivative and using the system (5), we have

$$\begin{aligned} \frac{dD(t)}{dt}& = c\widetilde{k}\int _{0}^{\infty } e^{-m_{2}\tau }f_{2}(\tau )\int _{0}^{\infty } e^{-m_{1}\tau }f_{1}(\tau ) \bigg (\lambda -d x(t-\tau )-\widetilde{\beta } x(t-\tau ) v(t-\tau )\bigg )d\tau d\tau \\
&\quad+\,c\widetilde{k}\int _{0}^{\infty } e^{-m_{2}\tau }f_{2}(\tau )\bigg (\widetilde{\beta }\int _{0}^{\infty } e^{-m_{1}\tau }f_{1}(\tau )\widetilde{\beta } x(t-\tau ) v(t-\tau )d\tau -a y(t)-\alpha w(t)y(t)\bigg )d\tau \\
&\quad+\,c\widetilde{k}\int _{0}^{\infty } e^{-m_{2}\tau }f_{2}(\tau )\bigg (a w(t)y(t)-b z(t)\bigg )d\tau + \frac{ac}{2} \bigg (\widetilde{k}\int _{0}^{\infty } e^{-m_{2}\tau }f_{2}(\tau )y(t)d\tau \\
&\quad -p v(t+\tau )\bigg )d\tau + \frac{b\widetilde{k}}{2}\int _{0}^{\infty } e^{-m_{2}\tau }f_{2}(\tau ) \bigg (c z(t)-q w(t)\bigg )d\tau \\
& = \lambda c\widetilde{k}\int _{0}^{\infty } e^{-m_{2}\tau}f_{2}(\tau )\int _{0}^{\infty } e^{-m_{1}\tau }f_{1}(\tau ) d\tau
d\tau -\bigg [dc \widetilde{k} \int _{0}^{\infty } e^{-m_{2}\tau
}f_{2}(\tau )\int _{0}^{\infty } e^{-m_{1}\tau }f_{1}(\tau
)\\
&\qquad x(t-\tau )d\tau d\tau + \frac{a}{2} c \widetilde{k}\int
_{0}^{\infty } e^{-m_{2}\tau }f_{2}(\tau ) y(t)d\tau +
\frac{b}{2}c\widetilde{k} \int _{0}^{\infty } e^{-m_{2}\tau
}f_{2}(\tau )z(t)d\tau \\
&\quad+\,q\frac{b \widetilde{k} }{2}\int _{0}^{\infty }
e^{-m_{2}\tau }f_{2}(\tau ) w(t)d\tau +p\frac{ac}{2}
v(t)\bigg ] \\
&\le \lambda c\widetilde{k}\int _{0}^{\infty } e^{-m_{2}\tau
}f_{2}(\tau )\int _{0}^{\infty } e^{-m_{1}\tau
}f_{1}(\tau ) d\tau d\tau \\
&\quad-\,\epsilon D(t) \left\{\begin{array}{ll} <0, & \text{ for } \
D(t)>\frac{\lambda c\widetilde{k}\int _{0}^{\infty }
e^{-m_{2}\tau }f_{2}(\tau )\int _{0}^{\infty } e^{-m_{1}\tau
}f_{1}(\tau ) d\tau d\tau }{\epsilon },\\
>0, & \hbox
{for}\ D(t)<\frac{\lambda c\widetilde{k}\int _{0}^{\infty }
e^{-m_{2}\tau }f_{2}(\tau )\int _{0}^{\infty } e^{-m_{1}\tau
}f_{1}(\tau ) d\tau d\tau }{\epsilon }. \\ \end{array} \right.
\end{aligned}$$

where $\epsilon =\min \{d, \frac{a}{2},\frac{b}{2},q,p\}$. This implies that *D*(*t*) is bounded. Thus all the solutions *x*(*t*), *y*(*t*), *v*(*t*), *z*(*t*) and *w*(*t*) are bounded.

In order to study the asymptotic behavior of the proposed model, we use Zaman (2011). The model (5) has three possible equilibria, disease-free equilibrium $E_0(x_{0},y_{0},z_{0},v_{0},w_{0})$, single-infection equilibrium $E_1(x_{1},y_{1},z_{1},v_{1},w_{1})$ and double-infection equilibrium $E_2(x_{2},y_{2},z_{2},v_{2},w_{2})$ which are given below,

$$\begin{aligned} E_{0}& = \left (\frac{\lambda }{d} , 0, 0, 0, 0 \right ), \\ E_{1}& = \bigg (\frac{ap}{ \widetilde{k}\widetilde{\beta } M_{1}M_{2}},\frac{\lambda \widetilde{\beta}\widetilde{k}M_{1}M_{2}-apd }{a\widetilde{\beta}\widetilde{k}}, 0,\frac{\lambda \widetilde{\beta } \widetilde{k}M_{1}M_{2}-apd }{a\widetilde{\beta } p} ,0\bigg ),\\ E_{2}& = \bigg (\frac{\lambda \alpha cp}{d\alpha cp+\widetilde{\beta } b \widetilde{k}q}, \frac{qb}{\alpha c}, \frac{q(\alpha \widetilde{\beta }\lambda c\widetilde{k} M_{1}M_{2}-\widetilde{\beta }ab \widetilde{k} q -a\alpha cdp)}{ac(\widetilde{\beta } b\widetilde{k} q+\alpha c dp)} , \frac{\widetilde{k} q b }{\alpha c p},\\&\qquad\frac{\widetilde{k} q b }{\alpha c p}, \frac{\alpha \widetilde{\beta }\lambda c\widetilde{k} M_{1}M_{2}-\widetilde{\beta } ab \widetilde{k} q -a\alpha cdp}{\alpha (\widetilde{\beta } b\widetilde{k} q+\alpha c dp)}\bigg ),\\ \end{aligned}$$

where $$ M_{i}=\int _{0}^{\infty } e^{-m_{i}\tau }f_{i}(\tau )d\tau , (i=1,2)$$.

The steady state with the pathogen presence is possible when the equilibrium density of the pathogen is greater than zero $(v_{1}> 0)$. This leads to a condition for invasion of the pathogen. Therefore, we can define

$$ R_0= \frac{\lambda \widetilde{\beta}\widetilde{k} \int _{0}^{\infty } e^{-m_{1}\tau }f_{1}(\tau )d\tau \int _{0}^{\infty } e^{-m_{2}\tau }f_{2}(\tau )d\tau }{apd }>0. $$

Here, $R_{0}$ is called the basic reproduction ratio of model which represents the average number of secondary virus produced from a single virus for system (5). Noting that $\int _{0}^{\infty } \tau f_{i}(\tau )d\tau =1$ and if $m_{i} > 0 (i = 1, 2)$, then $\int _{0}^{\infty } e^{-m_{i}\tau }f_{i}(\tau )d\tau < 1$. It is clear that increasing either of the delay may decrease the basic reproduction ratio $R_{0}$.

It turns out that the value of $R_{0}$ determines the existence of the single-infection equilibrium, that is $E_{1}$ exists if and only if $R_{0} > 1$. For the third equilibrium to exist, the density of the recombinant virus must be greater than zero $(w_{2} > 0)$ and this leads to the condition

$$ R_2= \frac{\alpha cdp}{\widetilde{\beta } b\widetilde{k}q }(R_{0}-1). $$

Hence, $$R_2 > 1$$ if and only if $$R_0 >R_{1} $$, where $$R_{1}= 1 + \frac{\widetilde{\beta } b\widetilde{k}q }{\alpha cdp}$$.

To analyze the stability of the equilibria, we need to calculate the characteristic equation of the Jacobian matrix of the system (5) at equilibrium point $E(\bar{x},\bar{y},\bar{z},\bar{v},\bar{w})$ as below

$$\begin{aligned} det[\eta I-J]=det \left( \begin{array}{ccccc} \eta +d+\widetilde{\beta }\bar{v} &\quad 0 &\quad 0 &\quad \widetilde{\beta } \bar{x} &\quad 0 \\ -\bar{v}\widetilde{\beta } N_{1}(\eta ) &\quad \eta +a+\alpha \bar{w} &\quad 0 &\quad -\bar{x}\widetilde{\beta } N_{1}(\eta ) &\quad \bar{y}\alpha \\ 0 &\quad -\bar{w}\alpha &\quad \eta +b &\quad 0 &\quad -\bar{y}\alpha \\ 0 &\quad -\widetilde{k} N_{2}(\eta ) &\quad 0 &\quad \eta +p &\quad 0 \\ 0 &\quad 0 &\quad -c &\quad 0 &\quad \eta +q \\ \end{array} \right) =0. \end{aligned}$$

where $$N_{i}(\eta )=\int _{0}^{\infty } e^{-m_{i}\tau }e^{-\eta \tau }f_{i}(\tau )d\tau , (i=1,2).$$

## Local behavior of the proposed model

In this section, we find the local stability of the system (5).

### **Theorem 2**

*When*$R_0 <1$, *then the disease-free equilibrium*$E_{0}$*is locally asymptotically stable while for*$R_0 >1$, $E_{0}$*becomes unstable and the single-infection equilibrium*$E_{1}$*occurs.*

### *Proof 2*

The characteristic equation of the Jacobian matrix of the linearized system corresponding to the system (5) at $E_{0}(\frac{\lambda }{d},0 ,0 ,0 ,0)$ is given by

$$ det[\eta I -J(E_{0})] =(b+\eta )(d+\eta )(q+\eta )\left[ (a+\eta )(p+\eta )-\frac{\lambda }{d}\widetilde{\beta } \widetilde{k} N_{1}(\eta )N_{2}(\eta ) \right] =0.$$

The three roots of the characteristic equation $\eta _{1}=-b$, $\eta _{2}=-d$ and $\eta _{3}=-q$ are negative and the remaining two roots are given by the following equation

7 $$ (a+\eta )(p+\eta )-\frac{\lambda }{d}\widetilde{\beta } \widetilde{k} N_{1}(\eta )N_{2}(\eta )=0. $$

Let us rewrite the above equation

8 $$ g(\eta )=(a+\eta )(p+\eta )-\frac{\lambda }{d}\widetilde{\beta }\widetilde{k} N_{1}(\eta )N_{2}(\eta ). $$

Noting that $$|N_{i}(\eta )|\le 1, (i=1,2)$$.

Let us assume $g(0)=ap(1-R_{0})<0$ and $\lim _{\eta \rightarrow \infty } g(\eta )=+\infty$. By the continuity of $g(\eta )$ there exist at least one positive root of $g(\eta )=0$. Thus, the infection-free equilibrium $E_0$ is unstable if $R_0 >1$.

If we choose the direct delta function $f_{i}(\tau )=\delta (t), (i=1,2)$ then we obtain $N_{i}(\eta )=1, (i=1,2).$ In this case Eq. (7) becomes

9 $$ \eta ^{2}+(a+p)\eta +ap(1-R_{0})=0.$$

Thus, if $R_{0}<1$, then Eq. (8) has two negative roots. Hence the equilibrium $E_{0}$ is locally asymptotically stable when $f_{i}(\tau )=\delta (t), (i=1,2)$.

If $i\nu (\nu > 0)$ is a solution of Eq. (7), it follows that

$$ -\nu ^{2} + (a +p)\nu i + ap -\widetilde{k}\widetilde{\beta } \frac{\lambda }{d}N_{1}(i\nu )N_{2}(i\nu ) = 0, $$

which yields

10 $$\nu ^{4} + (a^{2} +p^{2})\eta ^{2} + (ap)^{2} -\left(\widetilde{k}\widetilde{\beta } \frac{\lambda }{d}\right)^{2}|N_{1}(i\nu )|^{2}|N_{2}(i\nu )|^{2} =0. $$

We note that for $(i=1,2)$,

11 $$ |N_{i}(i\nu )|=\bigg |\int _{0}^{\infty } e^{-m_{i}\tau }\big (\cos (i\nu \tau )-\sin (i\nu \tau )\big )f_{i}(\tau )d\tau \bigg |\le \int _{0}^{\infty } e^{-m_{i}\tau }f_{i}(\tau )d\tau . $$

Therefore, we have

$$ (ap)^{2}-\left(\widetilde{k}\widetilde{\beta } \frac{\lambda }{d}\right)^{2}|N_{1}(i\nu )|^{2}|N_{2}(i\nu )|^{2}\ge (ap)^{2}(1-R_{0}^{2}). $$

Hence, if $R_{0}<1$, then Eq. (10) has no positive roots. Therefore, by the general theory on characteristic equations of delay differential equations (Kuang 1993) (Theorem 3.4.1), it is clear that $E_{0}$ is always locally asymptotically stable for $f_{i}(\tau )=\delta (t), (i=1,2)$, if $R_{0} < 1$.

### **Theorem 3**

*For*$1<R_0 <R_{1}$*the single infection-free equilibrium*$E_{1}$*is locally asymptotically stable, while*$E_{1}$*becomes unstable for*$R_0>R_{1}$*and recombinant virus may persist.*

### *Proof 3*

The characteristic equation corresponding to the Jacobian matrix of the linearized system of the system (5), is given by

$$\begin{aligned} det[\eta I - J(E_{1})]& = \big ((b+\eta )(q+\eta )-\alpha y_{1}c\big )\bigg [ \big (d+\widetilde{\beta } v_{1}+\eta )\widetilde{k}\widetilde{\beta } x_{1} N_{1}(\eta )N_{2}(\eta ) -(\widetilde{\beta })^{2}x_{1}kv_{1} N_{1}(\eta )N_{2}(\eta )\\&\quad-\,(d+\widetilde{\beta } v_{1}+\eta )(a+\eta )(p+\eta )\bigg ]=0. \end{aligned}$$

We can write the equation in the form $P_{1}(\eta )P_{2}(\eta )=0,$ where

$$\begin{aligned} P_{1}(\eta )& = (b+\eta )(q+\eta )-\alpha y_{1}c,\\ P_{2}(\eta )& = (d+\widetilde{\beta } v_{1}+\eta )\widetilde{k}\widetilde{\beta } x_{1} N_{1}(\eta )N_{2}(\eta )- (\widetilde{\beta })^{2}x_{1}\widetilde{k}v_{1}N_{1}(\eta )N_{2}(\eta )-(d+\widetilde{\beta } v_{1}+\eta )(a+\eta )(p+\eta ). \end{aligned}$$

Now $P_{1}(\eta )$ can be written as

$$P_{1}(\eta )=\eta ^{2}+(b+q)\eta +bq(1-R_{2}), $$

which shows that $P_{1}(\eta )=0$ has two roots with negative real parts iff $R_{2}<1$ (i,e $R_{0}<R_{1}$ or one positive and one negative if $R_{2}>1$ (i,e $R_{0}>R_{1}$, which shows that the single infection free equilibrium $E_{1}$ is unstable. $P_{2}(\eta )=0$ also can be written as

12 $$ \eta ^{3}+a_{2}(\tau )\eta ^{2}+a_{1}(\tau )\eta +a_{0}(\tau )+(b_{1}\eta +b_{2})N_{1}(\eta )N_{2}(\eta )=0, $$

where

$$\begin{aligned} a_{2}(\tau )& = a+p+d+\widetilde{\beta } v_{1},\\ a_{1}(\tau )& = (a+p)(d+\widetilde{\beta } v_{1})+ap,\\ a_{0}(\tau )& = ap(d+\widetilde{\beta } v_{1}),\\ b_{1}(\tau )& = -\widetilde{k}\widetilde{\beta } x_{1},\\ b_{0}(\tau )& = -d\widetilde{k}\widetilde{\beta } x_{1}. \end{aligned}$$

When $f_{i}(\tau )=\delta (\tau )$, we have $N_{i}(\eta )=1 (i=1,2)$. In this case Eq. (12) becomes

13 $$ \eta ^{3}+a_{2}(\tau )\eta ^{2}+(a_{1}(\tau )+b_{1}(\tau ))\eta +a_{0}(\tau )+b_{2}(\tau )=0, $$

By applying the Routh-Hurwitz criterion (Gantmacher 1959), we know that all the roots of (13) have negative real parts if $R_{0}>1$, because

$$\begin{aligned} a_{2}(\tau )& = a+p+d+d(R_{0}-1)>0, \\ a_{1}(\tau )+b_{1}(\tau )& = (a+p)(d+d(R_{0}-1)>0, \\ a_{0}(\tau )+b_{2}(\tau )& = apd(R_{0}-1)>0. \end{aligned}$$

Finally, we have

$$ a_{2}(a_{1}+b_{1})-(a_{0}+b_{2}) = \big (dR_{0}\big (a^{2}+(a+p)(p+dR_{0}\big )+apd\big )>0.$$

Therefore, the equilibrium $E_{1}$ is locally asymptotically stable when $f_{i}(\tau )=\delta (\tau ) (i=1,2)$.

If $i\nu$ for $\nu >0$ is a solution of Eq. (13), then it follows that

14 $$ -i\nu ^{3}+a_{2}(\tau )\nu ^{2}+a_{1}(\tau )i\nu +a_{0}(\tau )+(b_{1}i\nu +b_{2})N_{1}(i\nu )N_{2}(i\nu )=0.$$

After some simplification, we get

15 $$ \nu ^{6}+(a_{2}^{2}-2a_{1})\nu ^{4}+(a_{1}^{2}-2a_{0}a_{2})\nu ^{2}+a_{0}^{2}-(b_{2}^{2}+b_{1}^{2}\nu ^{2})|N_{1}(i\nu )|^{2}|N_{2}(i\nu )|^{2}=0, $$

where

$$\begin{aligned} a_{2}^{2}-2a_{1}& = (d+\widetilde{\beta }v_{1})^{2}+a^{2}+p^{2}>0,\\ a_{1}^{2}-2a_{0}a_{2}-b_{1}^{2}|N_{1}(i\nu )|^{2}|N_{2}(i\nu )|^{2}& = (d+\beta v_{1})^{2}(a^{2}+p^{2})+a^{2}p^{2}-(\widetilde{k}\widetilde{\beta } x_{1})|N_{1}(i\nu )|^{2}|N_{2}(i\nu )|^{2} \\&\ge (a^{2}+p^{2})(d+\widetilde{\beta } v_{1})^{2},\\ (a_{0}^{2}-b_{2}^{2})|N_{1}(i\nu )|^{2}|N_{2}(i\nu )|^{2}& = (a^{2}+p^{2})(ap(d+\widetilde{\beta } v_{1}))^{2}-(d\widetilde{k}\widetilde{\beta } x_{1})^{2}|N_{1}(i\nu )|^{2}|N_{2}(i\nu )|^{2}\\&\ge ap\widetilde{\beta } v_{1}\bigg [ap(d+\widetilde{\beta } v_{1})+dk\widetilde{\beta } x_{1}|N_{1}(i\nu )|^{2}|N_{2}(i\nu )|^{2}\bigg ]. \end{aligned}$$

Hence if $R_{0}>1,$ then the Eq. (15) has no positive roots. So by the general theory of characteristic equations of delay differential equations (Kuang 1993), the chronic infection equilibrium $E_{1}$ is locally asymptotically stable when $f_{i}(\tau )=\delta (\tau ), (i=1,2)$.

## Global behavior of the proposed model

In this section, we study the global behavior of the system (5). To do this we will use Lyapunove functionals theory and Lasali’s invariance principle.

### **Theorem 4**

*The disease-free equilibrium*$E_{0}$*is globally asymptotically stable when*$R_0 <1$.

### *Proof 4*

Let $\big (x(t),y(t),z(t),v(t),w(t)\big )$ be any positive solution of the system (5) with initial conditions (6). Consider the Lyapunove functional

16 $$ L_{E_{0}}(t)=L_{1}(t)+L_{2}(t), $$

where

$$\begin{aligned} L_{1}(t)& = x(t)-x_{0}-\ln \frac{x(t)}{x_{0}}+r_{1}y(t)+r_{1}z(t)+r_{2}v(t)+r_{1}\frac{b}{c}w(t),\\ L_{2}(t)& = r_{1}\widetilde{\beta } \int _{0}^{\infty } f_{1}(\tau )e^{-m_{1}\tau }\int _{t-\tau }^{t}x(\theta ) v(\theta )d\theta d\tau +r_{2}\widetilde{k} \int _{0}^{\infty }f_{2}(\tau )e^{-m_{2}\tau }\times \int _{t-\tau }^{t}y(\theta ) d\theta d\tau \end{aligned}$$

with $$r_{1}=\frac{1}{\int _{0}^{\infty } e^{-m_{1}\tau }f_{1}(\tau )d\tau }$$ and $$r_{2}=\frac{1}{\widetilde{k}\int _{0}^{\infty } e^{-m_{1}\tau }f_{1}(\tau )d\tau \int _{0}^{\infty } e^{-m_{2}\tau }f_{2}(\tau )d\tau }. $$

By taking derivative of $L_{1}(t)$ along the positive solution of the system (5), we have

$$\begin{aligned} \frac{dL_{1}(t)}{dt}& = \left(x(t)-\frac{x}{x_{0}}\right)\bigg (\lambda -d x(t)-\widetilde{\beta } x(t) v(t)\bigg )+r_{1}\bigg (\widetilde{\beta } \int _{0}^{\infty } f_{1}(\tau )e^{-m_{1}\tau } x(t-\tau )v(t-\tau )d\tau \\&\quad-\,a y(t)-\alpha w(t)y(t) \bigg )+r_{1} \bigg (a w(t)y(t)-b z(t)\bigg ) +r_{2}\bigg (\widetilde{k} \int _{0}^{\infty }f_{2}(\tau )e^{-m_{2}\tau }y(t-\tau )d\tau -p v(t)\bigg )\\&\quad+\,r_{1}\frac{b}{c}\bigg (c z(t)-q w(t)\bigg ). \end{aligned}$$

On substituting $\lambda =dx_{0}$, and simplifying, we get

17 $$\begin{aligned} \frac{dL_{1}(t)}{dt}& = \frac{(x(t)-x_{0})^{2}}{x}-\widetilde{\beta } x(t) v(t)+r_{1}\bigg (\widetilde{\beta } \int _{0}^{\infty } f_{1}(\tau )e^{-m_{1}\tau } x(t-\tau )v(t-\tau )d\tau  \\&\quad-\,a y(t)-\alpha w(t)y(t) \bigg )+r_{1} \bigg (a w(t)y(t)-b z(t)\bigg ) +r_{2}\widetilde{k}\bigg ( \int _{0}^{\infty }f_{2}(\tau ) \\&\quad\times\,e^{-m_{2}\tau }y(t-\tau )d\tau -p v(t)\bigg )+r_{1}\frac{b}{c}\bigg (c z(t)-q w(t)\bigg ), \\& = \frac{-d(x(t)-x_{0})^{2}}{x}-\widetilde{\beta }x(t) v(t)+r_{1}\bigg (\widetilde{\beta } \int _{0}^{\infty } f_{1}(\tau )e^{-m_{1}\tau } x(t-\tau )v(t-\tau )d\tau\bigg)  \\&\quad-\,r_{1}a y(t) +r_{2}\widetilde{k} \int _{0}^{\infty }f_{2}(\tau )e^{-m_{2}\tau }y(t-\tau )d\tau -r_{1}\frac{bq}{c }w(t)+r_{2}p(R_{0}-1)v(t) \\&\quad-\,r_{1}\frac{bq}{c}w(t). \end{aligned}$$

By taking the derivative of $L_{2}(t)$, we get

18 $$\begin{aligned} \frac{dL_{2}(t)}{dt}& = r_{1}\widetilde{\beta } \int _{0}^{\infty } f_{1}(\tau )e^{-m_{1}\tau }\bigg (x(t)v(t)-x(t-\tau )v(t-\tau )\bigg )d\tau +r_{2}\widetilde{k} \int _{0}^{\infty }f_{2}(\tau )e^{-m_{2}\tau } \\&\quad\times\,\bigg (y(t)-y(t-\tau \bigg )d\tau . \end{aligned}$$

Taking derivative of Eq. (16) and using Eqs. (17) and (18) and simplifying, we get

19 $$\begin{aligned} \frac{dL_{E_{0}}(t)}{dt}& = -d\frac{(x(t)-x_{0})^{2}}{x}+\frac{p(R_{0}-1)v(t)}{\widetilde{k}\int _{0}^{\infty } f_{1}(\tau )e^{-m_{1}\tau }d\tau \int _{0}^{\infty } f_{2}(\tau )e^{-m_{2}\tau }d\tau }-r_{1}\frac{bq}{c}w(t). \\ \end{aligned}$$

If $R_{0}\le 1$, it follows from Eq. (19) that $\frac{d}{dt}V_{E_{0}}(t)\le 0$. Moreover, the equality also holds if $x_{0}=\frac{\lambda }{d}$, $y(t)=0$, $z(t)=0$, $v(t)=0$, $w(t)=0$. Hence by LaSalle’s invariance principle (see LaSalle (1976)), we conclude that $E_{0}$ is globally asymptotically stable when $R_{0}<1$.

### **Theorem 5**

*If*$1<R_{0}<R_{1}$, *then the single infection free equilibrium*$E_{1}$*is globally asymptotically stable, implying that the recombinant virus cannot survive but the pathogen virus can exist.*

### *Proof 5*

Let (*x*(*t*), *y*(*t*), *z*(*t*), *v*(*t*), *w*(*t*)) be any positive solution of the system (5) with initial conditions (6). Let us consider the Lyapunove functional

20 $$ V_{1}(t)= \big (x(t)-x_{1}\ln x(t)\big )+r_{1}\big (y(t)-y_{1}\ln y(t)\big )+r_{2}\big (v(t)-\ln v(t)\big )+r_{1}z(t)+r_{1}\frac{b}{c}w(t), $$

where $r_{1}$ and $r_{2}$ are discussed in the previous theorem. Now taking derivative of $V_{1}(t)$, we get

$$\begin{aligned} \frac{dV_{1}(t)}{dt}& = \left(1-\frac{x_{1}}{x}\right)\bigg (\lambda -d x(t)-\widetilde{\beta } x(t) v(t)\bigg )+r_{1}\left(1-\frac{y_{1}}{y}\right)\bigg (\widetilde{\beta }\int _{0}^{\infty } e^{-m_{1}\tau }f_{1}(\tau )d\tau x(t-\tau ) v(t-\tau )\\&\quad-\,a y(t)-\alpha w(t)y(t)\bigg )+r_{1}\bigg (a w(t)y(t)-b z(t)\bigg )\\&\quad+\,r_{2}\left(1-\frac{v_{1}}{v}\right)\bigg (\widetilde{k}\int _{0}^{\infty } e^{-m_{2}\tau }f_{2}(\tau ) y(t-\tau )d\tau -p v(t)\bigg )+r_{1}\frac{b}{c}\bigg ( c z(t)-q w(t)\bigg ).\\ \end{aligned}$$

Using $\lambda =d x_{1}-\widetilde{\beta } x_{1}v_{1}$ in the above equation, we have

21 $$\begin{aligned} \frac{dV_{1}(t)}{dt}& = -\frac{(x(t)-x_{1})^{2}}{x}-\widetilde{\beta } x(t)v(t)+\widetilde{\beta } x_{1}v_{1}\bigg (1-\frac{x_{1}}{x}\bigg )+r_{1}\widetilde{\beta }\int _{0}^{\infty } e^{-m\tau }f_{1}(\tau )d\tau x(t-\tau )d\tau  \\&\quad-\,r_{1}\frac{\widetilde{\beta } y_{1}}{y}\int _{0}^{\infty } e^{-m_{1}\tau }f_{1}(\tau )d\tau x(t-\tau ) v(t-\tau )v(t-\tau )d\tau -r_{1}ay(t) \\&\quad+\,\widetilde{\beta } x_{1}v_{1}+r_{2}\widetilde{k}\int _{0}^{\infty } e^{-m_{2}\tau }f_{2}(\tau )d\tau y(t-\tau )-r_{2}\widetilde{k} \frac{v_{1}}{v(t)}\int _{0}^{\infty } e^{-m_{2}\tau }f_{2}(\tau ) y(t-\tau )d\tau  \\&\quad+\,\widetilde{\beta }x_{1}v_{1}+r_{1}(\alpha y_{1}-\frac{bq}{c})w(t). \end{aligned}$$

Let us define

22 $$\begin{aligned} V_{E_{1}}(t)& = V_{1}(t)+r_{1}\widetilde{\beta }\int _{0}^{\infty } e^{-m_{1}\tau }f_{1}(\tau )\int _{t-\tau }^{t}\bigg [x(\rho )v(\rho )-x_{1}v_{1}-xv\ln \frac{x(\rho )v(\rho )}{x_{1}v_{1}}\bigg ]d\rho d\tau  \\&\quad+\,r_{2}\widetilde{k}\int _{0}^{\infty } e^{-m_{2}\tau }f_{2}(\tau )\int _{t-\tau }^{t}\bigg [y(\rho )-y_{1}-y_{1}\ln \frac{y(\rho )}{y_{1}}\bigg ]d\rho d\tau . \end{aligned}$$

Taking derivative of Eq. (22) and using Eq. (21), we get

23 $$\begin{aligned} \frac{d V_{E_{1}}(t)}{dt}& = -\frac{(x(t)-x_{1})^{2}}{x}-\widetilde{\beta } x(t)v(t)+\widetilde{\beta } x_{1}v_{1}\bigg (1-\frac{x_{1}}{x}\bigg )+r_{1}\widetilde{\beta }\int _{0}^{\infty } e^{-m_{1}\tau }f_{1}(\tau )d\tau x(t-\tau )d\tau  \\&\quad-\,r_{1}\frac{\widetilde{\beta } y_{1}}{y}\int _{0}^{\infty } e^{-m_{1}\tau }f_{1}(\tau ) x(t-\tau ) v(t-\tau )d\tau -r_{1}ay(t) \\&\quad+\,\widetilde{\beta } x_{1}v_{1}+r_{2}\widetilde{k}\int _{0}^{\infty } e^{-\tau }f_{2}(\tau )d\tau y(t-\tau )-r_{2}\widetilde{k} \frac{v_{1}}{v(t)}\int _{0}^{\infty } e^{-m_{2}\tau }f_{2}(\tau ) y(t-\tau )d\tau  \\&\quad+\,\widetilde{\beta } x_{1}v_{1}+r_{1}\left(\alpha y_{1}-\frac{bq}{c}\right)w(t)+r_{1}\widetilde{\beta }\int _{0}^{\infty } e^{-m_{1}\tau }f_{1}(\tau )\bigg [x(t)v(t)-x(t-\tau )v(t-\tau ) \\&\quad+\,x_{1}v_{1}\ln \frac{x(t-\tau )v(t-\tau )}{x(t)v(t)}\bigg ]d\rho d\tau +r_{2}\widetilde{k}\int _{0}^{\infty } e^{-m_{2}\tau }f_{2}(\tau )\bigg [y(t)-y(t-\tau )+y_{1}\ln \frac{y(t-\tau )}{y(t)}\bigg ] \\& = -\frac{(x(t)-x_{1})^{2}}{x}+\widetilde{\beta } x_{1}v_{1}\bigg (1-\frac{x_{1}}{x}\bigg )-r_{1}\widetilde{\beta }\int _{0}^{\infty } e^{-m_{1}\tau }f_{1}(\tau )\ln \frac{x(t-\tau )v(t-\tau )}{x_{1}v_{1}y(t)}d\tau +\widetilde{\beta } x_{1}v_{1} \\&\quad-\,r_{2}\widetilde{k}y_{1}\int _{0}^{\infty } e^{-m_{2}\tau }f_{2}(\tau )\ln \frac{v_{1}y(t-\tau )}{y_{1}v(t)}d\tau +\widetilde{\beta } x_{1}v_{1}-r_{1}\widetilde{\beta }\int _{0}^{\infty } e^{-m_{1}\tau }f_{1}(\tau ) \\&\quad\times\,\ln \frac{x(t-\tau )v(t-\tau )}{x(t)v(t)}d\tau +r_{2}\widetilde{k}y_{1}\int _{0}^{\infty } e^{-m_{2}\tau }f_{2}(\tau )\ln \frac{y(t-\tau )}{y(t)}d\tau +r_{1}\left(\alpha y_{1}-\frac{bq}{c}\right)w(t), \\& = -\frac{(x(t)-x_{1})^{2}}{x}+\widetilde{\beta } x_{1}v_{1}\bigg (1-\frac{x_{1}}{x}-\frac{x}{x_{1}}\bigg )-r_{1}\widetilde{\beta } x_{1}v_{1}\int _{0}^{\infty } e^{-m_{1}\tau }f_{1}(\tau )\bigg [\frac{y_{1}x(t-\tau )v(t-\tau )}{x_{1}v_{1}y(t)} \\&\quad-\,1 -\ln \frac{y_{1}x(t-\tau )v(t-\tau )}{x_{1}v_{1}y(t)}\bigg ]d\tau -r_{2}\widetilde{k}y_{1}\int _{0}^{\infty } e^{-m_{2}\tau }f_{2}(\tau )\bigg [\frac{v_{1}y(t-\tau )}{y_{1}v(t)}-1-\ln \frac{v_{1}y(t-\tau )}{y_{1}v(t)}\bigg ]d\tau  \\&\quad-\,\frac{\alpha r_{1}p d }{\widetilde{\beta } \widetilde{k}}(R_{1}-R_{0})w(t). \end{aligned}$$

If $$R_{0} \le R_{1}$$, it follows from Eq. (23) that $$\frac{dV_{E_{1}}(t)}{dt}\le 0$$ for $$x_{1},y_{1},v_{1}>0$$. Also equality holds when $$x=x_{1}$$ and $$y=y_{1}$$, $$ v=v_{1}$$, $$ z=0$$ and $$w=0$$. Thus the solutions limit to the largest invariant subset of $${\frac{dV_{E_{1}}}{dt}=0}$$. Then, by LaSalle’s invariance principle (LaSalle 1976), we conclude that $$E_{1}$$ is globally asymptotically stable. This complete the proof.

## Numerical simulation

**Fig. 1:**
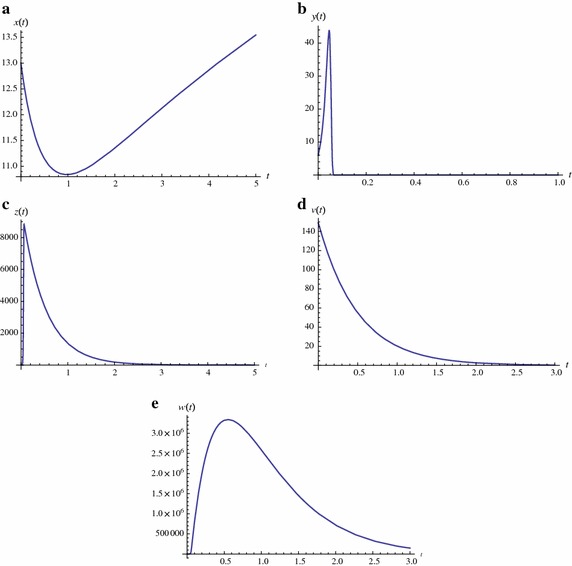
Stimulated time history when $\beta = 0.004, \lambda = 2, d = 1/10, \alpha = 0.004, a = 1/2, p = 2, k = 50, b = 2, c = 2000, m_1 = 1/2, m_2 = 1/2, q = 2$ with initial conditions $x(0)=13, y(0)=6, z(0)=3, v(0)=149, w(0)=1$

In this section, we present numerical simulation. We use Runge–Kutta order four method to find numerical results. For our numerical simulation we used parameters values $$\beta = 0.004$$ (estimated), $$ \lambda = 2$$ (Philips 1996), $$ d = 1/10$$ (Philips 1996), $$ \alpha = 0.004$$, (estimated), $$ a = 1/2$$ (estimated), $$p = 2$$ (Philips 1996), $$k = 50$$ (Hass 1999), $$b = 2$$ (Schnell et al. 1997), $$c = 2000$$ (Schnell et al. 1997), $$m_1 = 1/2= m_2 = 1/2$$ (assumed), $$q = 2$$ (assumed) with initial conditions $$x(0)=13, y(0)=6, z(0)=3, v(0)=149, w(0)=1.$$ Our numerical results show that, by using continuous delays in latent and virus production periods, then the number of healthy cells increases and the virus load reduces as shown by Figure 1a and d, respectively. Figure 1b shows that the number of infected cells decreases due to reducing the viral load by postponing the production period of infected cells. Figure 1d shows that the number of double infected cells increases which release the recombinant virus to fight with pathogens virus. Figure 1e the shows that recombinant virus are decreasing with the passage of time.

## Conclusion and discussion

In this work, we presented the asymptotic analysis of an HIV-1 epidemic model by incorporating distributed intracellular delays. Our one delay term used for latent period and the second one used for virus production period. From the corresponding characteristic equations, it was shown that if the basic reproduction ratio is less than unity, the infection-free equilibrium is locally asymptotically stable. We also proved that the chronic-infection equilibrium exists and is locally asymptotically stable if the basic reproduction ratio is greater than one. Similarly, the global stability of the infection-free equilibrium and the chronic-infection equilibrium of the proposed model have been completely established under certain conditions. It is clear from these results that intracellular delays describing the latent period and viral production period have great effect on the stability of feasible equilibria and therefore, do not induce periodic oscillations. Numerical results of our proposed model represented that continuous delays in latent period and virus production period can help in reducing the load of pathogen virus due which the number of infected cells reduced and CD4+ cells are increased.
